# Environmental Gradients Shape the Distribution of Free-Living and Host-Associated Syndiniales Life Stages

**DOI:** 10.1007/s00248-026-02831-1

**Published:** 2026-07-08

**Authors:** Neea Hanström, Kinlan M. G. Jan, Monika Winder

**Affiliations:** Department of Ecology, Environment, and Plant Sciences, Stockholm University, Svante Arrhenius väg 20 A, Stockholm, 114 18 Sweden

**Keywords:** Syndiniales, Dinospore Distribution, Protists, Parasites, Spatial Variation

## Abstract

Marine alveolate parasites, particularly early-branching dinoflagellates of the order Syndiniales, play critical yet often overlooked roles in shaping marine microbial communities. These parasitoids infect diverse hosts, including dinoflagellates, copepods, and fish eggs, and rely on short-lived, free-living dinospores for transmission. Despite their ecological significance, the distributions of Syndiniales dinospores and host-associated life stages across environmental gradients remain poorly understood. We used 18S rRNA gene region DNA metabarcoding combined with size-fractionated water filtration across vertical and horizontal gradients of salinity, oxygen, and nutrients in the Baltic Sea-Skagerrak system to characterize Syndiniales life-stage distributions and identify clades producing dinospores. This approach enables the differentiation of free-living dinospores and host-associated life stages. Most reads were assigned to Syndiniales groups I and II, indicating the presence of both host associations and dinospore presence. Dinospore communities were more diverse than host-associated life stages and showed variation in spatial distribution and community composition. The spatial variation was related to salinity, oxygen, and nitrogen concentrations, emphasizing the role of environmental conditions in shaping niches suitable for host-parasite associations and infection transmission. Our findings highlight the prevalence of Syndiniales communities across an environmental gradient and the influence of environmental conditions on their distribution patterns.

**Supplementary Information:**

The online version contains supplementary material available at 10.1007/s00248-026-02831-1.

## Introduction

Alveolata constitute the dominant protist parasites in marine environments, with early-branching dinoflagellates in the order Syndiniales being particularly prevalent [21]. Global molecular studies confirm their substantial contribution to microbial communities [15, 30]. Syndiniales are parasitoids, meaning they eventually kill their hosts to complete their life cycle. They infect a wide variety of hosts, including other dinoflagellates [6], ciliates [25], copepods [52], and fish eggs [51, 56]. Syndiniales are divided into five groups (SG), which are further divided into clades (e.g., C-4). Some well-characterized species have been described, such as *Ichthyodinium chabelardi* (SG I), *Amoebophyra* species complex (SG II), *Syndinium turbo* (SG IV), and *Hematodinium perezi* (SG IV). The taxonomy of many clades is still under investigation, and numerous taxa have been detected only in environmental samples [3], indicating that the full host range needs further exploration. Host associations have increasingly been explored using metabarcoding approaches [26, 42, 68], and Syndiniales life-cycle strategies are described in a few taxa [13]. However, water-sample filtration with a single filter size combined with metabarcoding approaches does not distinguish free-living dinospores from host-associated stages, leaving the distribution patterns of the different life stages largely unresolved.

Dinoflagellate parasitoids have a free-living dispersal life stage known as dinospores or zoospores [11]. Syndiniales life cycles are described for a few taxa, such as the SG II *Amoebophyra* species complex [6, 13]. The free-swimming, biflagellate dinospore, measuring about 3–10 μm, serves as the primary infective form that locates and infects new hosts [6, 53]. Infection occurs through penetration of the host surface [52] or ingestion of dinospores [24]. Transmission has also been suggested to occur through secondary intake via infected prey items acting as transmission vectors [20]. Following infection, the invasive growth and feeding stage develops and transforms into a sac-like trophont that differs in overall appearance from typical dinoflagellates. The life cycle concludes in a reproductive phase in which the organism undergoes sporogenesis, producing large numbers of new dinospores [52, 54, 55]. Dinospores are released either as individual dinospores or as a worm-like vermiform structure (aggregation of multiple dinospores) reaching up to 20 μm [9, 36], both of which are released into the water through host lysis. Once released into the water, the vermiform stages disintegrate into individual dinospores. Dinospores are short-lived and must typically locate a new host within 48 h to continue their life cycle [11]. The full infection cycle can be completed within 24–72 h [19]. However, some parasites have developed strategies to survive periods of host scarcity by forming vegetative resting cysts in their hosts or in the sediment [6, 64].

Shared environmental preference between hosts and parasites create co-occurrence patterns that increase encounter rates. The structure of the Syndiniales community composition is strongly influenced by environmental gradients, such as depth, location, and abiotic conditions [20], yet limited seasonal variation has been observed [2]. Environmental conditions, in addition to host traits, such as feeding behaviour, influence parasite-host associations [12, 41]. Parasitic associations have high potential to affect ecosystem functioning through several mechanisms. Infections can alter host population dynamics and host distributions, with consequences for trophic interactions. For example, parasites can terminate phytoplankton blooms [7, 34, 63] or favor certain species by infecting their grazers or competitors [27, 57]. Some Syndiniales have been linked to the collapse of toxic red-tide dinoflagellate populations [7, 36], while the lack of parasites has been suggested to prolong bloom duration [34, 35]. A similar pattern has also been suggested in the Baltic Sea, as the nanoflagellate community fluctuations are reported to be related to short-lived bloom events [45]. Syndiniales associations with cercozoans and diatoms are reported [3, 48], and their impact on langoustine fisheries is well documented [29]. Moreover, case studies show that some parasitic dinoflagellates can cause mortality and reduced fecundity in copepods [27, 66]. Syndiniales host population dynamics are well-established in the environment, and the potentially detrimental impacts of infections are described for many hosts. However, the community composition and distribution patterns of dispersal life stages, and the transmission of Syndiniales parasites in the environment require further investigation.

In this study, we explored the distribution patterns of the free-living and host-associated Syndiniales life stages along the brackish-marine environmental continuum. We used DNA metabarcoding targeting the V4 region of the 18S rRNA gene for samples collected at three depth strata along the horizontal and vertical environmental gradients. Water samples were size-fractionated to capture mostly the free-living stages in the pico-nanoplankton fraction (0.2–20 μm) and the host-associated life stages and resting cysts in the microplankton size fraction (> 20 μm) [28, 48]. Syndiniales clades detected in both filter size fractions may represent actively reproducing parasites associated with diverse phytoplankton and microzooplankton hosts. In contrast, clades detected exclusively in the pico-nano fraction predominantly represent free-living dinospore stage. They likely infect hosts smaller than 20 μm that passed through the larger filter, or larger organisms, such as mesozooplankton, which are not effectively captured by small-volume water filtration. We hypothesize that dinospore distribution patterns vary across depth strata and sampling locations, depending on the environmental conditions that promote host-parasite co-occurrence. To identify associations between the Alveolata and some of the most abundant zooplankton taxa, we also investigated associations between Syndiniales and copepod hosts.

## Materials and Methods

### Sampling

**Fig. 1:**
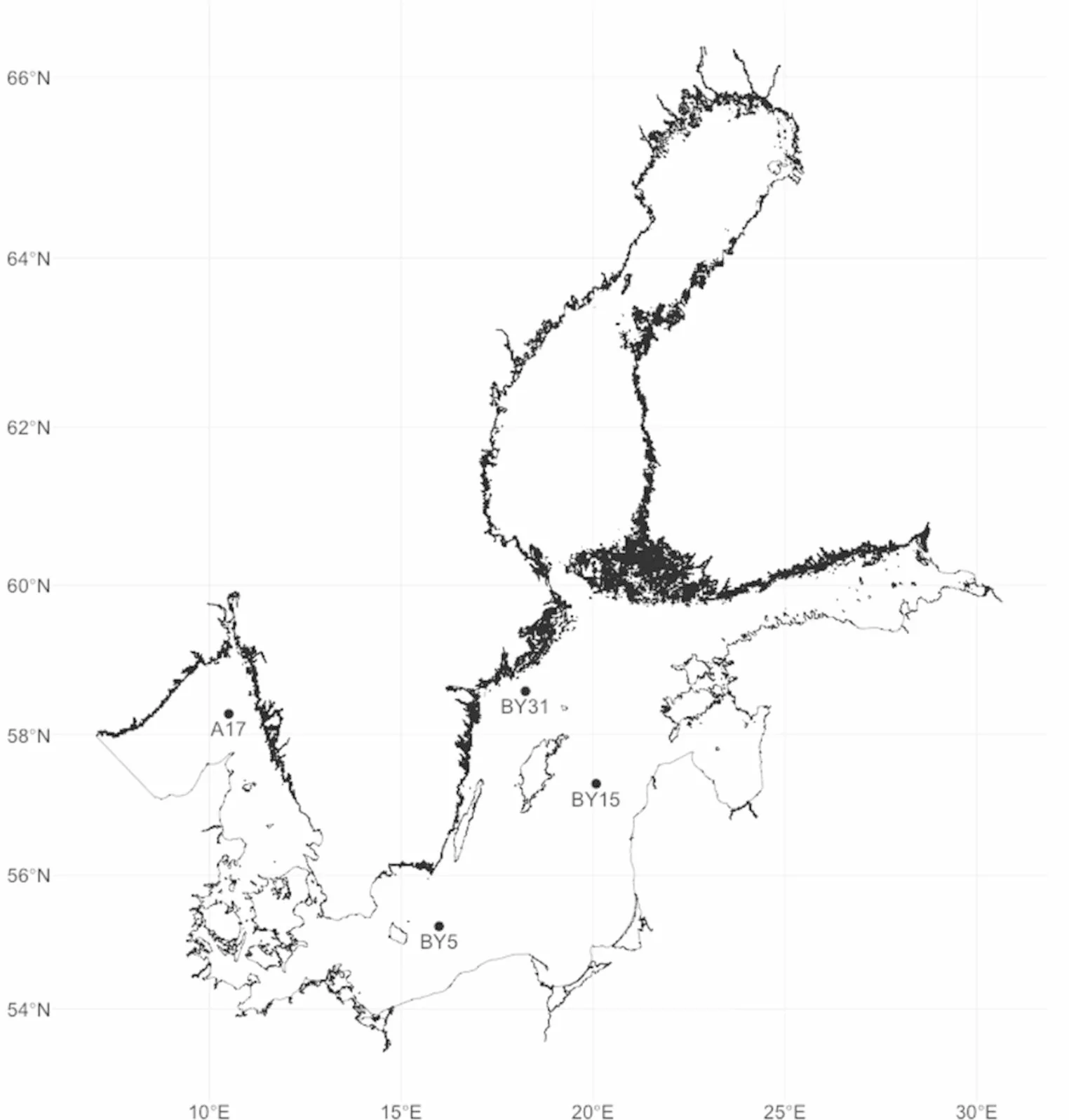
Map of the Baltic Sea with the four sampling locations BY31, BY15, BY5, and A17. Coordinate data for map creation is retrieved from ICES (2005)

Samples were collected in August 2023 from four locations along the Baltic Sea-Skagerrak environmental gradient, representing different sub-basins of the Baltic Sea (Fig. 1). Temperature, salinity, oxygen, phosphate, nitrate, and chlorophyll-*a* concentrations were measured at each sampling location across the water column (Fig. S1) and retrieved from SMHI [58]. Water samples were collected from the surface to 90 m depth with Niskin bottles at varying intervals. Equal aliquots from different depths within the surface (0–30 m), medium (30–60 m), and deep (60–90 m) strata were mixed to obtain two replicates of a 1 L sample for each depth stratum, which were filtered on board using a peristaltic pump (Masterflex L/S). We used 25 mm diameter polycarbonate filters with pore sizes of 20 μm and 0.2 μm to capture microplankton (> 20 μm) and pico- and nanoplankton (0.2–20 μm) size fractions, respectively. The filters were stored at −20 °C for two to three months until DNA extraction.

We collected zooplankton samples with vertical hauls using a WP2 closing net with 90 μm mesh size from the same three depth strata as the water samples at each sampling location. Zooplankton was preserved in 70% ethanol and stored at −20 °C until sorting. Zooplankton species sorted for DNA metabarcoding included the copepods *Centropages* spp. (including *C. hamatus* and *C. typicus*), and *Pseudocalanus* spp. (including *Pseudocalanus acuspes* and *Pseudocalanus elongatus)* from all locations, and additionally, *Calanus* spp. in the marine sampling location A17. From each sample, three replicates consisting of five individuals per copepod genus were hand-picked and rinsed for 30 s in a 1% bleach bath to remove externally attached symbiont DNA, as described by Zamora-Terol et al., [67].

### DNA Extraction, PCR, and Sequencing

DNA was extracted from all the samples with a QIAmp DNA Micro Kit (Qiagen). We PCR amplified the 18S V4 gene region encoding SSU rRNA. We used a universal eukaryotic primer pair covering high dinoflagellate species diversity from environmental samples (SSU556F: 5′-CGCGGTAATTCCAGCTYC-3′, and SSU911R: 5′-ATYCAAGAATTTCACCTCTGAC-3′) [59]. PCR reactions contained 10 µL of KAPA HiFi HotStart ReadyMix (Roche, KAPA Biosystems), 1 pmol of each primer, 6 µL of molecular-grade water, and 2 µL of extracted DNA per sample (varying concentrations). PCR conditions consisted of an initial denaturation at 98 $\:^\circ\:$C for 2 min, followed by 35 cycles of denaturation for 30 s at 98 $\:^\circ\:$C, annealing for 30 s at 64 $\:^\circ\:$C, and an extension for 1 min at 72 $\:^\circ\:$C. The final elongation was completed with 5 min at 72 $\:^\circ\:$C, and then the PCR product was stored at 4 $\:^\circ\:$C. Sequencing libraries were subsequently constructed using the PCR product as template as described by Novotny et al., [37].

### Bioinformatics and Data Analysis

Processing of raw fastq data was facilitated using the nf-core/ampliseq pipeline (v. 2.11.0) [16, 60]. Primers were removed with cutadapt [31]. Amplicon sequence variants (ASVs) were inferred using the DADA2 package [5] in R [47]. Sequences were trimmed before median quality scores dropped below 25 while retaining at least 75% of the reads, resulting in trimming of forward reads at 282 bp and reverse reads at 231 bp. Reads shorter than these were discarded. Taxonomy assignment was performed against the PR^2^ database (v. 5.0.0;) [18].

Only Alveolata reads were retained in the dataset, and samples with fewer than 100 Alveolata reads were excluded from further analysis. Rarefaction curves showed a steep increase and a plateau for all samples (Fig. S2), indicating adequate sequencing effort. Statistical analysis and visualization of the results were performed using R packages phyloseq [32] and tidyverse core packages [65]. Venn diagrams of Syndiniales ASVs across locations, filter sizes, and depth strata were produced using the VennDiagram R package [8]. ASV richness of Syndiniales was defined as the number of unique ASVs per sample at each location. Community diversity was assessed with the Shannon index (H′) and Gini-Simpson index (1 − D), capturing both richness and evenness, as well as dominance, using the vegan package [39]. All indices were calculated for each sample according to location, depth strata, and filter size. Differences in the mean ASV richness, Shannon, and Gini-Simpson (1 – D) indices across locations, depth strata, and filter sizes were tested with the Kruskal–Wallis test, followed by Dunn’s post-hoc test with Benjamini–Hochberg (BH) adjustment to control for multiple testing.

Community composition patterns were explored using principal coordinates analysis (PCoA) based on Bray-Curtis dissimilarities using the vegan package in R [39]. Differences in community composition across locations, depth strata, and filter sizes were tested using PERMANOVA (pseudo-F statistics obtained through 999 permutations). All water samples were first tested together, and then PERMANOVAs were conducted separately for the two filter-size classes. Parameters were tested while accounting for marginal effects to ensure each factor was tested independently. The resulting pseudo-F, the coefficient of determination R², and *p*-values were used to evaluate the relative importance and significance of each factor. We used the envfit function to correlate environmental gradients, including salinity, temperature, oxygen, chloropyll-*a*, and nitrate and phosphate concentrations, with the ordination axes with 999 permutations. As envfit was used to explore correlations between environmental gradients and ordination structure rather than to infer independent effects, collinear variables were retained.

## Results

### Sequencing Output and Community Composition

In total, 1,310,688 reads were sequenced. After quality filtration, a total of 508,947 reads were retained, of which 419,610 were from 41 water samples (Table S1) and 89,337 reads from 28 copepod samples. Dinoflagellate reads accounted for 346,616 reads in the water samples and 56,301 reads in the zooplankton samples, representing altogether almost 80% of all Alveolata reads, followed by Ciliata (18.7%), and Perkinsea (2.1%) (Table S2). The relative proportions differed between water and copepod samples, as dinoflagellates represented 82.5% and 63% of the reads, and ciliates for 14.9% and 36.5%, respectively (Table S2).

**Fig. 2:**
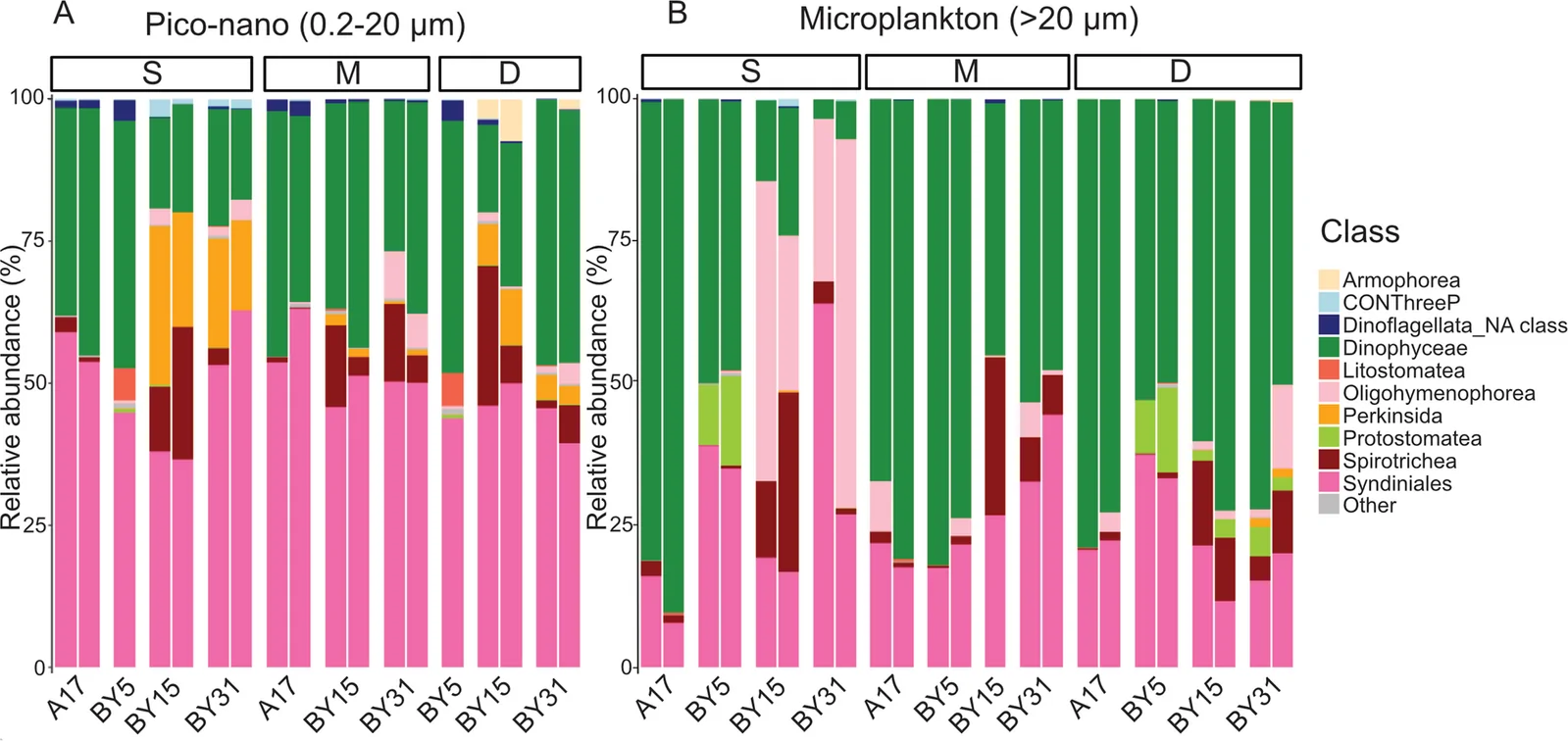
Relative read abundance of Alveolata classes in the (**A**) pico-nano (0.2–20 μm) and (**B**) microplankton (> 20 μm) size fractions for each sample in the different depth strata (S = surface, M = medium, and D = deep) and locations (x-axis). The ten Alveolata classes with the highest relative abundance are presented in colour, while the remaining Alveolata classes are presented in grey (Other)

The proportions of the different Alveolata classes varied among water samples, with Syndiniales accounting for about 35% of the Alveolata reads on average across all locations. Syndiniales represented approximately half of the reads (49%) in the pico-nano size fraction, but only about 25% on average in the microplankton fraction (Fig. 2). In both filter fractions, Dinophyceae (47%) and Oligohymenophorea (22%) were also dominant classes within the Alveolata reads across all water samples. The ciliate classes Oligohymenophorea and Spirotrichea were most abundant in the low salinity locations BY15 and BY31 in the surface strata, where they represented around one-third of the reads on average.

### ASV Richness and Spatial Distributions

**Fig. 3:**
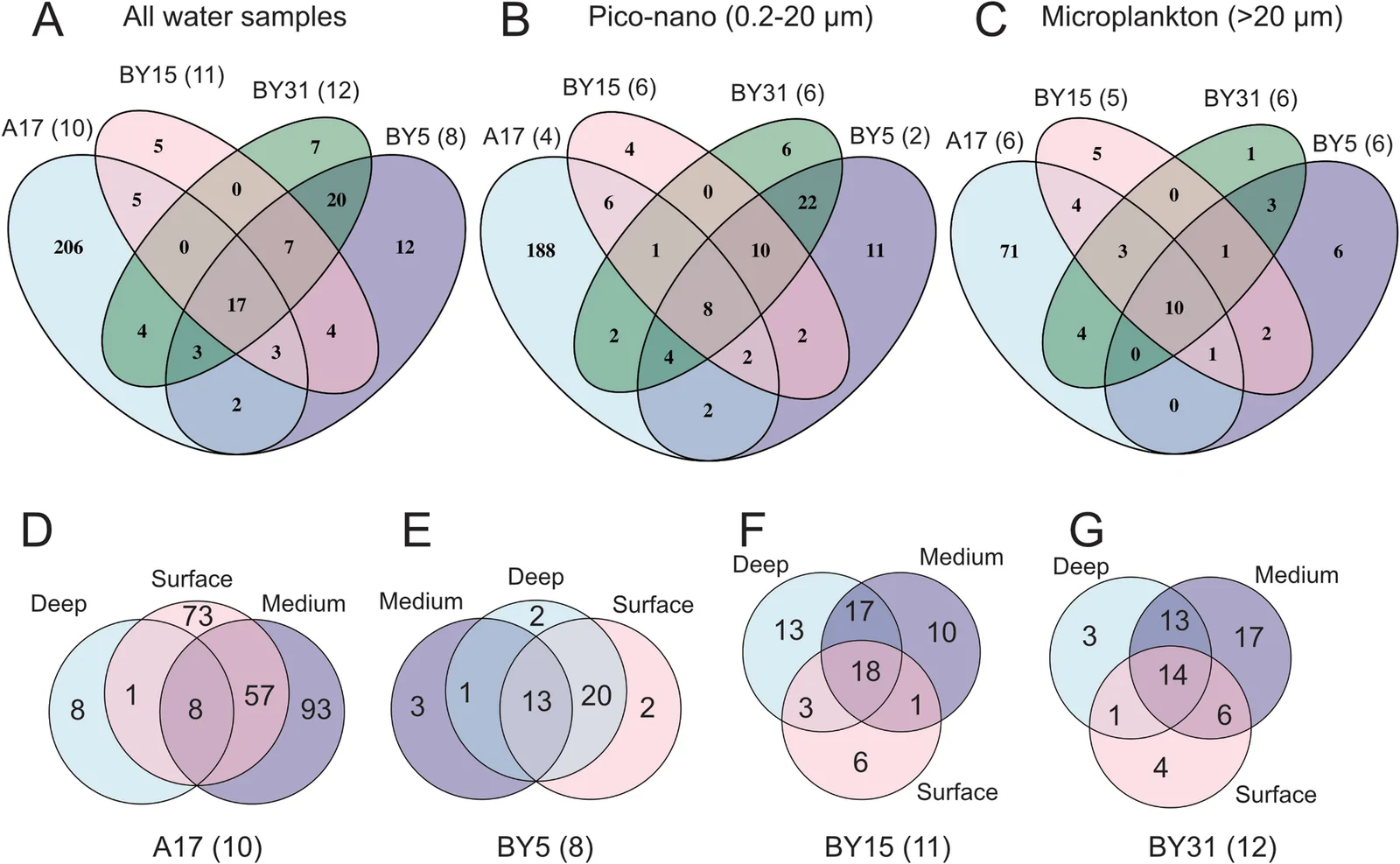
Number of shared and unique Syndiniales ASVs in the water column (A) of the pico-nano (0.2-20 µm) and microplankton (>20 µm filter size fractions, (B) of the pico-nano (0.2-20 µm) and (C) of the microplankton (>20 µm) filter size fractions across the sampling locations, and (D-G) in each location across depth strata and both filter sizes. The number of samples included in each panel is presented in brackets after the location name (see details in Table S1).

The number of Syndiniales ASVs retrieved from the water samples varied among locations, with 17 ASVs shared between all locations (Fig. 3). The total number of all Syndiniales ASVs was highest at A17 (240), compared to BY5 (41), BY15 (68), and BY31 (58). Likewise, the number of unique ASVs was higher in the marine Skagerrak location A17 (206) as compared to the Baltic Sea locations BY5 (12), BY15 (5), and BY31 (7) (Fig. 3). Mean ASV richness was highest at A17 (58.0 ± 40.6 SD, *n* = 10) and lower in the Baltic Sea locations BY15 (20.5 ± 11.6, *n* = 11), BY31 (17.3 ± 11.0, *n* = 12), and BY5 (17.5 ± 7.6, *n* = 8). Median ASV richness differed significantly among locations (Kruskal–Wallis: chi-sq = 10.549, df = 3, *p* = 0.014), with higher richness in the Skagerrak A17 compared to the Baltic Sea locations (Dunn’s post-hoc: Z = 2.31–3.06, *p* = 0.042) with no significant differences among Baltic Sea locations (Fig. S3, Table S3). The pico-nano fraction generally contained more ASVs than the microplankton fraction, except in BY15. Only 8 ASVs in the pico-nano and 10 ASVs in the microplankton fraction were shared across all locations (Fig. 3B–C). Depth-related patterns varied among locations (Fig. 3D–G), with the highest number of unique ASVs at the medium stratum in A17 (93), while Baltic Sea locations showed greater overlap among depth strata.

Differences in Shannon diversity (H′) were observed between size fractions (Kruskal-Wallis: chi-sq = 7.322, df = 1, *p* = 0.007), with pico-nano size fraction generally showing higher Shannon values (Fig. S4). Differences among locations were evident, with A17 exhibiting the highest overall Shannon diversity, while the Baltic Sea locations consistently showed lower values (Kruskal-Wallis: chi-sq = 14.493, df = 3, *p* = 0.002; Dunn’s post hoc: BY5: Z = 3.331, *p* = 0.004; BY15: Z = 2.307, *p* = 0.042; BY31: Z = 3.243, *p* = 0.004). Differences in the diversity were observed among the Baltic Sea locations’ depth strata (Kruskal-Wallis: chi-sq = 8.362, df = 2, *p* = 0.015), surface and deep (Dunn’s post hoc: Z = −2.744, *p* = 0.018). Similarly, the Gini-Simpson index varied among locations (Kruskal-Wallis: chi-sq = 13.455, df = 3, *p* = 0.004) and size fractions (Kruskal-Wallis: chi-sq = 11.843, df = 1, *p* < 0.001). Higher Gini-Simpson index values were observed in the microplankton filter samples across locations (Fig. S4). Gini-Simpson index values were lower at A17 than at the Baltic Sea locations BY31 (Dunn’s post hoc: Z = −3.246, *p* = 0.006) and BY5 (Dunn’s post hoc: Z = −3.084, *p* = 0.006), which showed higher Gini-Simpson index values, indicating a lower degree of taxonomic dominance across samples.

### Syndiniales Clade Composition Across Size Fractions

**Fig. 4:**
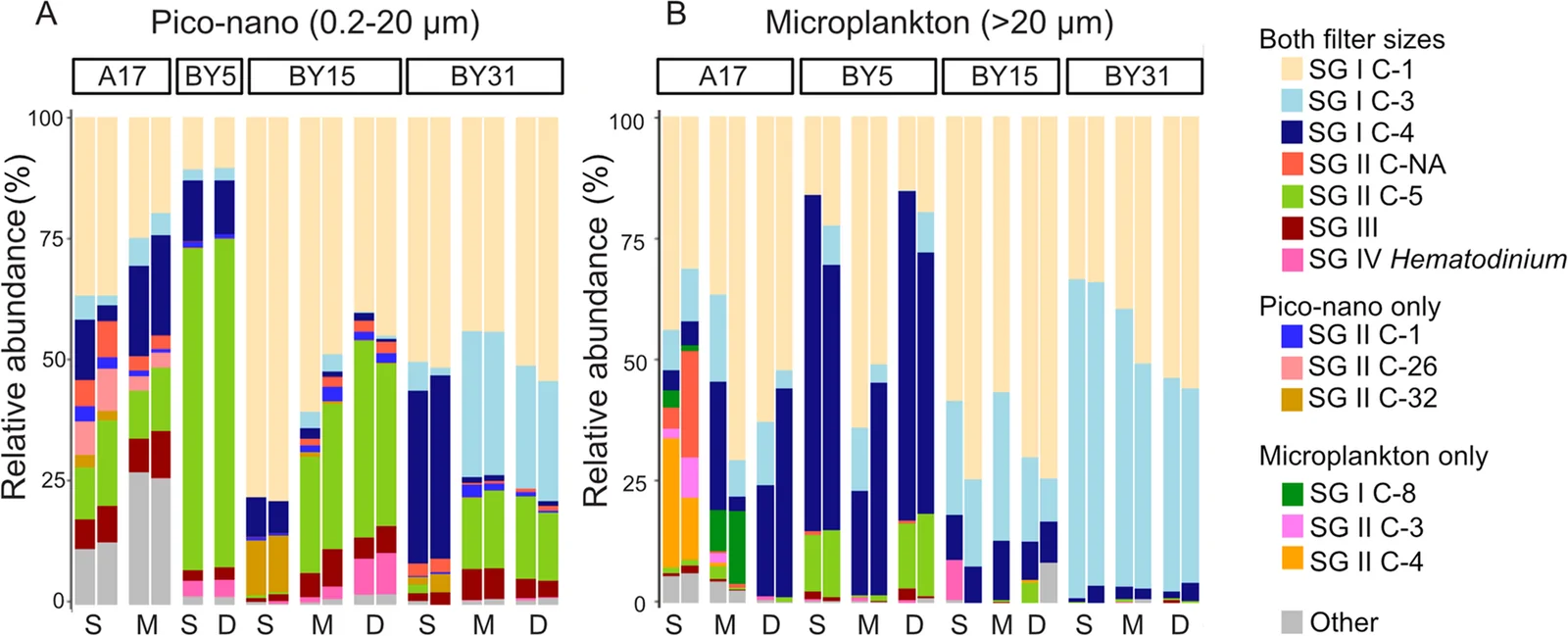
Relative read abundance of Syndiniales clades in the (**A**) pico-nano (0.2–20 μm) and (**B**) microplankton (> 20 μm) size fractions. The ten clades with the highest relative read abundance are presented in colour, while the “Other” includes all the remaining Syndiniales clades in grey. The letters on the x-axis stand for the sampling depth strata (S = surface, M = medium, and D = deep)

In both size fractions, Syndiniales were predominantly assigned to SGs I and II (Fig. 4, Fig. S5). SG I C-1 was the dominant clade, representing on average 43% of all Syndiniales reads. Seven of the 10 clades with the highest relative read abundance were shared between both size fractions. Several clades exhibited distinct size-related distribution patterns. SG I C-4 was more abundant in the microplankton fraction, accounting for an average of 20% of reads, but only 10% in the pico-nano fraction. In contrast, SG II C-5 was on average around tenfold more abundant in the pico-nano fraction than in the microplankton fraction across samples.

**Fig. 5:**
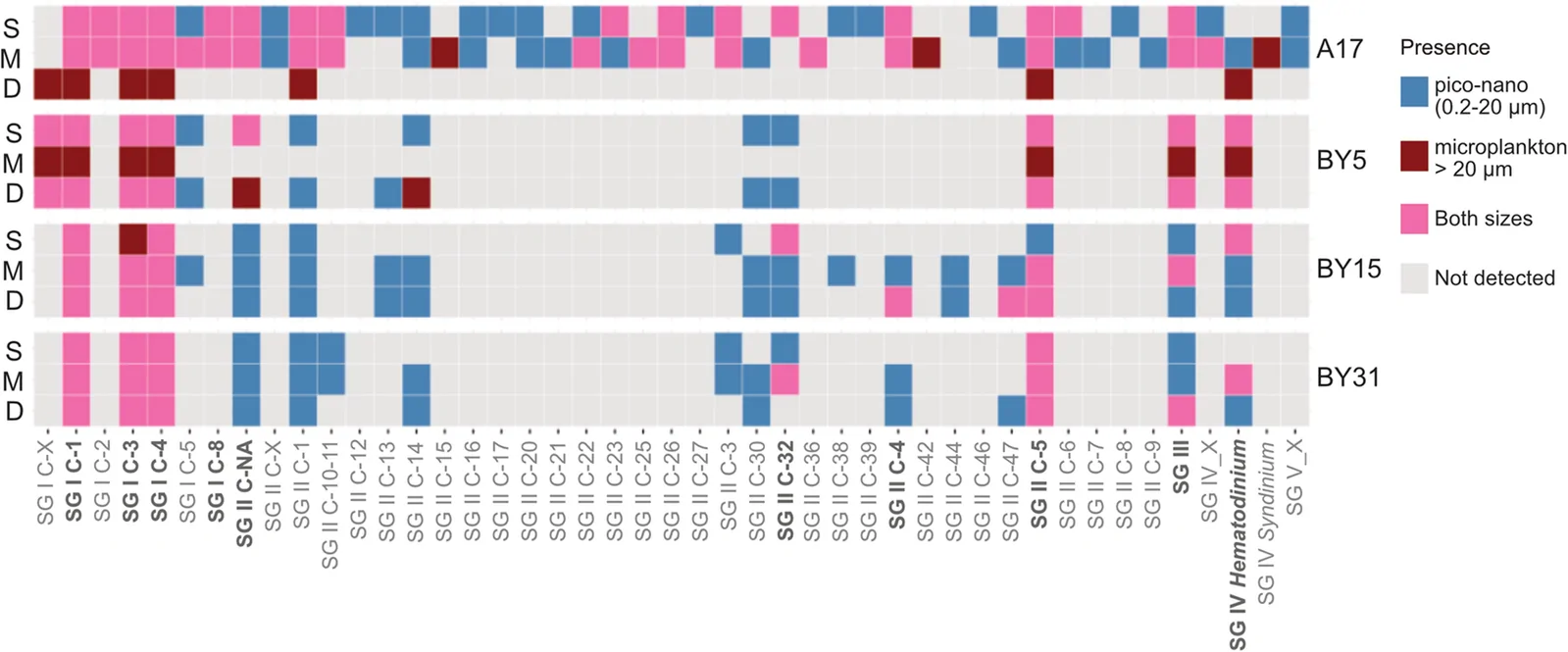
Unique and shared Syndiniales clades between the pico-nano (0.2–20 μm) and microplankton (> 20 μm) size fractions across the depth strata at each sampling location. The letters on the y-axis stand for the depth strata (S = surface, M = medium, and D = deep). The clades contributing to the ten clades with the highest relative read abundance on average across all water samples are highlighted in bold. In A17 deep and BY15 medium, only the microplankton filter size fraction passed the quality control

Syndiniales clade community composition varied among locations and depth strata. At BY31, the microplankton fraction was strongly dominated by SG I C-1 and C-3, which together comprised over 90% of the reads, whereas the pico-nano fraction showed higher clade diversity. At BY5, SG I C-4 dominated the microplankton fraction (52%), while SG II C-5 dominated the pico-nano fraction (67% on average). At BY15, SG II C-32 was among the 10 most abundant clades in surface pico-nano samples but was not among the dominant clades in the microplankton fraction (Fig. 4). However, it was still detected in both size fractions (Fig. 5). In deep-water pico-nano samples from BY15, SG IV *Hematodinium* represented 8% of the Syndiniales reads, whereas it occurred at lower relative abundances in other locations (1.2% on average; Fig. S5) and was not detected in surface samples from BY31 and A17 (Fig. 5). Several clades were exclusively detected in the pico-nano fraction at specific locations and depth strata (Fig. 5). Many of these clades belonged to SG II and were detected only in A17. Notably, SG V was also observed exclusively in the pico–nano fraction at the A17 location (Fig. 5).

**Fig. 6:**
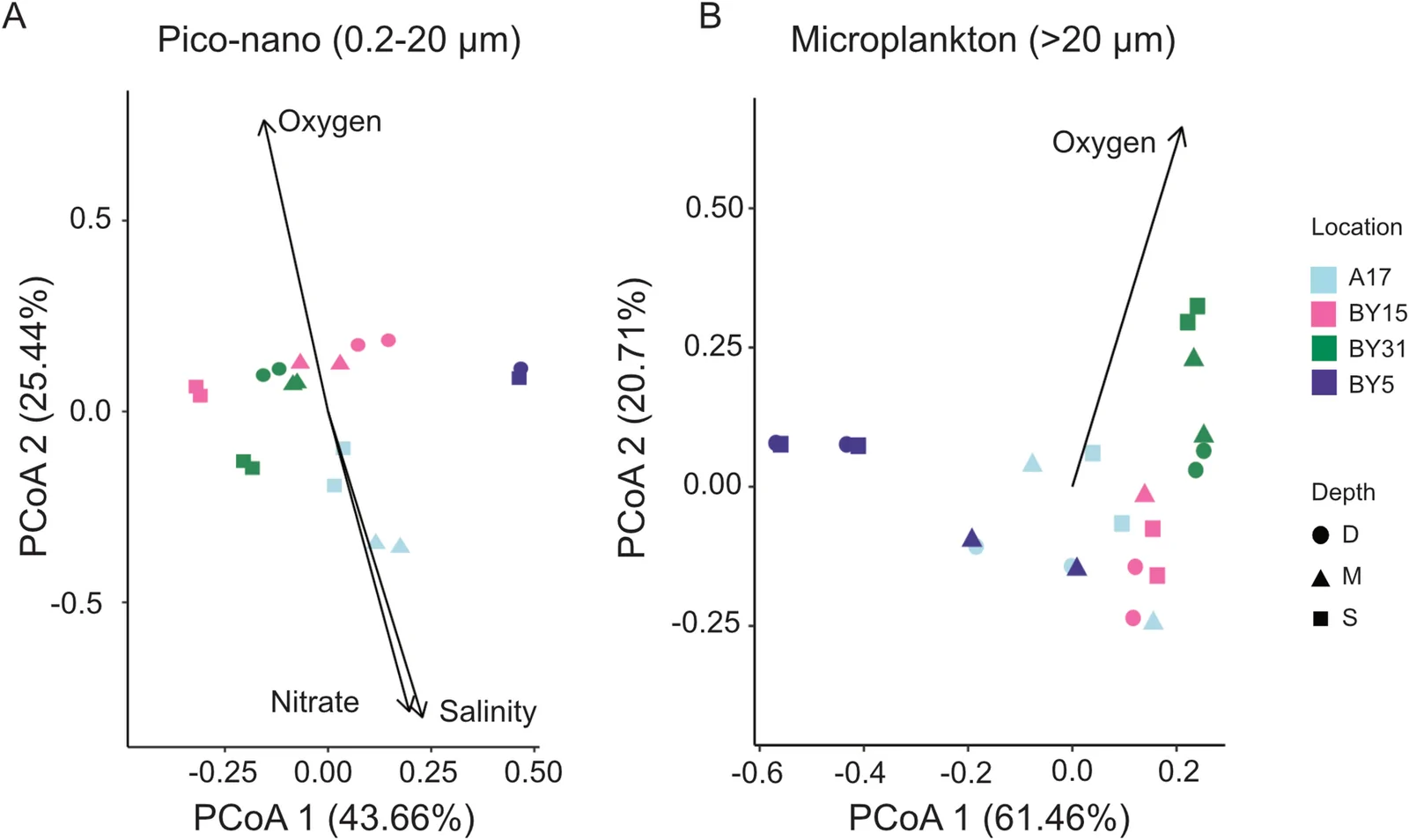
PCoA based on Bray-Curtis dissimilarity of the Syndiniales clades in the water samples across the locations and sampling strata for the (**A**) pico-nano size and (**B**) microplankton size fractions. The significantly related environmental parameters are indicated with the arrows fitted with the envfit function. The shapes marked with letters indicate the sampling strata: S = Surface, M = Medium, and D = Deep

The differences in Syndiniales clade community composition across all water samples were primarily associated with size fraction and sampling location. Marginal PERMANOVA revealed that location (R² = 0.42, *p* < 0.001) and size fraction (R² = 0.18, *p* < 0.001) explained most of the variation in community composition, whereas depth strata captured only about 3% of the variation (Table S4). When tested separately by size fraction, both sampling location (pico-nano: R² = 0.59, *p* < 0.001; microplankton: R² = 0.67, *p* < 0.001) and depth strata (pico-nano: R² = 0.15, *p* = 0.002; microplankton: R² = 0.09, *p* = 0.022) had significant effects on community composition within each size fraction (Table S4). The PCoA showed clustering by location, especially within the microplankton fraction in BY15 and BY31 (Fig. S6). Environmental fitting analyses showed that salinity, oxygen, and nitrate were significantly correlated with community composition in the pico-nano fraction (R^2^ ≥ 0.61, *p* = 0.011). In the microplankton fraction, only oxygen correlated significantly with the ordination (R² = 0.46, *p* = 0.018) (Fig. 6, Table S5).

**Fig. 7:**
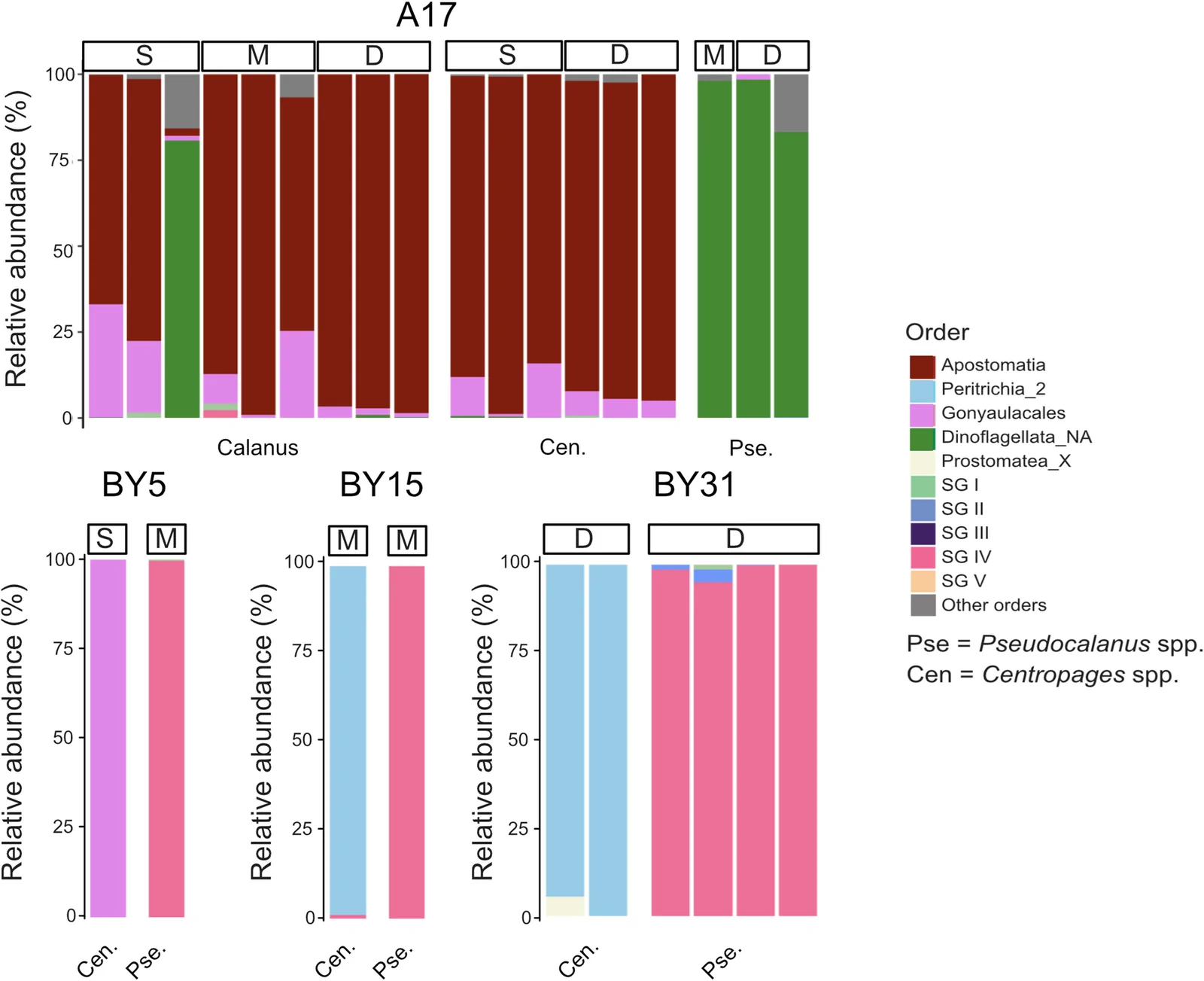
The relative read abundance of the Alveolata orders based on 18S within the copepod host taxa in the different locations across all depth strata: *Pseudocalanus* spp. (Pse.), *Centropages* spp. (Cen.) and *Calanus* spp. Five most abundant Alveolata orders, and the different Syndiniales groups, are shown in different colours. All other Alveolata orders are grouped under “Other orders” in grey. The letters indicate the sampling strata: S = surface, M = medium, and D = deep

The copepod *Pseudocalanus* spp. was mainly associated with SG IV at depths below 30 m across all locations except A17 (Fig. 7). In A17, all the copepod hosts were primarily associated with the ectoparasitic ciliate group Apostomatia (class Oligohymenophorea within Alveolata), which accounted for an average of 64% of reads. Unidentified dinoflagellate reads were also abundant at A17, accounting for up to 70% of reads in *Pseudocalanus* spp. *Centropages* spp. showed location-specific differences in associated taxa, with Apostomatia reaching the highest relative abundance in A17, Gonyaulacales in BY5, and Peritrichia in BY15 and BY31(Fig. 7).

## Discussion

In this study, we investigated the distribution patterns and environmental drivers of free-living dinospores and host-associated life stages of the parasitic Syndiniales. The Syndiniales community showed higher ASV diversity at A17 compared to the low-salinity locations in the Baltic Sea. The community structure at A17 was characterized by increased richness. This pattern was particularly pronounced in the pico-nano size fraction between A17 and Baltic Sea locations, suggesting a more diverse free-living dinospore community at higher salinity. Similar patterns have been observed in the Baltic Sea, as the number of Syndiniales ASVs was reported to increase at higher salinities in the Gulf of Gdansk [44]. Syndiniales are widespread throughout the Baltic Sea system [22], and our results highlight their prevalence despite differences in diversity, as they accounted for about half of all Alveolata reads across the salinity gradient. High Syndiniales relative read abundance is also consistent with global-scale studies demonstrating their widespread occurrence and ecological importance, particularly in pico-nanoplankton fractions [15, 28, 35, 48]. Across locations and size fractions, the community was dominated by SGs I and II, with the most abundant clades detected in both filter-size fractions. This overlap suggests the simultaneous presence of host-associated stages and free-living dinospores, indicating that the dominant clades likely maintain active life cycles characterized by continuous release of dinospores into the water column.

### Syndiniales-Host Associations Across Salinity Gradients

Dinophyceae include many putative hosts for Syndiniales, and their relative abundances were high in the microplankton fraction, especially at high-salinity location A17 and depths below 30 m. A high relative abundance of Dinophyceae (around 80%), together with a pronounced relative abundance of Syndiniales belonging to the *Amoebophyra* species complex (SG II C-4), a well-established intracellular parasite of dinoflagellates [13, 63], was observed at A17 surface. The co-occurrence of Syndiniales and Dinophyceae may indicate favourable conditions for host-parasite associations that could affect dinoflagellate population dynamics. In contrast, low-salinity surface waters in BY31 and BY15 showed reduced relative abundance of Dinophyceae and increased relative abundance of Perkinsida, in addition to ciliates belonging to Oligohymenophorea and Spirotrichea. Perkinsida are commonly associated with brackish and freshwater systems and parasitize a broad range of protists, including dinoflagellates [54]. Along with environmental drivers, such as low nutrient availability and the high abundance of zooplankton grazers in the Baltic Sea in August [23], dinoflagellate-infecting Perkinsida may partly explain the lower relative abundance of Dinophyceae in low-salinity environments. Additionally, we observed a high relative abundance of Syndiniales taxa known to parasitize ciliates, such as SG I C-4 (genus *Eudubosquella*) [10, 25]. Together with previous evidence that ciliates graze on Syndiniales dinospores [24], these observations suggest a complex network of interactions in which Perkinsida and Syndiniales infect both Dinophyceae and ciliates. The resulting release of dinospores into the water column may further facilitate parasite transmission while also providing prey for grazers.

### Syndiniales Associations between Filter-Size Fractions

The majority of the Syndiniales clades with high relative abundance were present in both the microplankton and pico-nano size fractions, indicating the presence of host-associated and free-living dinospores in the water column [28]. SG I C-1 and C-3 were among the most abundant clades in both size fractions. SG I C-3 includes the well-described parasite *Ichthyodinium chabelardi*, which infects fish eggs and causes mortality in newly hatched larvae [33, 43, 56]. The dinospores of *I. chabelardi* range from 6 –15 μm to 20–25 μm, depending on the stage of spore division [56], which likely explains its detection in both size fractions. In the Baltic Sea, fish eggs of Atlantic cod (*Gadus morhua*) and sprat (*Sprattus sprattus balticus*) may represent potential hosts for *Ichthyodinium*, as their spawning periods extend until August [4, 38]. Another highly abundant clade, SG II C-5, was also present in both size fractions, representing nearly one-third of Syndiniales reads on average across samples. However, its taxonomy is not fully determined, and its host associations are unknown. Similarly, SG III was among the abundant clades and was detected in both size fractions, although its relative read abundance remained on average low (< 10%), with higher relative abundance in the pico-nano size fraction. Previous studies reported correlations between SG III and dinoflagellates and diatoms [3], suggesting possible associations with these taxa in our samples, where Dinophyceae dominated the Alveolata community.

Only a few Syndiniales clades were detected exclusively in the microplankton size fraction, each contributing less than 5% of reads across samples. These clades, such as SG II C15 and 42, are likely associated with hosts that are retained in the larger size fraction during filtration, including ciliates and dinoflagellates. However, their absence from the pico-nano fraction suggests limited or undetectable dinospore release into the water column and potentially a dormant stage within the host [6]. Host abundance may also be low, since even small increases in host availability have been shown to significantly increase dinospore release into the water column [1]. Dinoflagellate abundances are typically low in the Baltic Sea during summer months [23], and therefore, the full range of host-associated life stages may not have been captured by our sampling. In addition, the possibility of a recent host population collapse following a parasitic outbreak cannot be ruled out, as nanoplanktonic flagellates (2–20 μm) in the Baltic Sea have been associated with short-lived bloom events [45]. Nevertheless, size-fractionated filtration is a widely used and robust approach for assessing plankton biodiversity across different size classes [15, 48, 61], even with lower replication [17, 50].

Some Syndiniales clades were detected only in the pico-nano size fraction, presumably representing free-living dinospores [35]. Syndiniales clade composition was more diverse in the pico-nano size fraction than in the microplankton fraction, following the previously observed patterns in size-fractionated sampling [15, 28]. Although previous studies suggested that reads in the pico-nano size fraction are unlikely to originate from debris derived from larger cells [28], the possibility of cross-contamination between size fractions through cell breakage cannot be completely excluded [48]. The pico-nano size fraction also includes reads from life stages associated with host organisms smaller than 20 μm. Many clades detected only in the pico-nano fraction belonged to SG II, but SG V was detected only at the A17 location in this fraction. SG V has only recently been added to the taxonomic resolution of Syndiniales, and, similar to SG III, its ecological associations require further investigation. The hosts of clades detected only in the pico-nano size fraction may include taxa that are not efficiently sampled by water filtration, explaining the non-detection of these clades in the microplankton size fraction. Such hosts may include large protists (> 35 μm) with patchy distributions and low abundance [40, 48] or mesozooplankton, such as copepods.

### Alveolata Associations with Copepods

At A17, most copepods were associated with a high relative abundance of the ectoparasitic ciliate group Apostomatia, known parasites of mesozooplankton [46, 49]. In the Baltic Sea locations, *Pseudocalanus* spp. were strongly associated with SG IV *Hematodinium*, consistent with previous observations [20]. In the water samples, SG IV *Hematodinium* was present only in the pico-nano size fraction in deeper strata at BY15 and BY31. These findings suggest dinospore production at these locations, suggesting ongoing infection and reproduction, likely involving copepod hosts, as associations between *Hematodinium* and copepods have previously been reported in deep water environments [20]. In contrast, *Centropages* spp. were primarily associated with ciliates and dinoflagellates. These patterns indicate specific associations between copepod hosts and different Alveolata classes in specific environmental conditions. Investigating the impacts of these parasite-host associations on host fitness could provide important insights into unresolved microbial pathways in food web functioning, including the role of host lysis and increased vulnerability of infected copepods to predation.

### Environmental Gradients Shape Syndiniales Community Composition

The results show that environments with higher salinity and nitrate but lower oxygen concentrations were linked to distinct Syndiniales clade composition in the pico-nano fraction. These environmental conditions are typical for the Baltic Sea deep waters below the halocline. Several environmental variables, including salinity, oxygen, nutrients, and temperature, were intercorrelated, reflecting an environmental gradient rather than independent drivers. High relative abundance of Syndiniales reads in the pico-nano size fraction suggests that these conditions may favour active dinospore production driven by infection in the presence of hosts. Overall, these findings suggest environmental niche specificity, in which environmental conditions shape host and parasite community composition across horizontal and vertical gradients during summer. Previous studies showed that Syndiniales community composition changes along salinity fluctuations, and that not all ASVs are present at salinities below 7 [44], indicating limitations in transmission under low salinity. Successful infection likely depends on suitable conditions for host-parasite co-occurrence and sufficient host density, which regulates encounter rates and facilitates parasite transmission [1]. The global-scale impacts of environmental conditions on protist community composition have been observed [28], and our results further suggest that environmental conditions structure host-parasite interactions and shape Baltic Sea protist communities. Since dinospores are short-lived outside the host [11], unfavourable environmental conditions for transmission, such as hypoxia or low salinity [14], might limit parasite transmission. However, the role of hypoxia in parasite survival and transmission remains unexplored, even though some Syndiniales clades are reported to tolerate hypoxic conditions [42, 62].

## Conclusions

Our findings highlight the widespread occurrence of Syndiniales across spatial scales, demonstrating their ubiquity in both brackish and marine habitats. Through their ability to shape species interactions and community dynamics [6, 36], Syndiniales prevalence suggests an important role in ecosystem functioning across environmental gradients. The results suggest that most Syndiniales clades maintain active life cycles, characterized by dinospore release into the water column and host-associated stages linked to ciliates, Dinophyceae, and other potential host taxa. Certain clades, such as SG IV *Hematodinium*, interact with copepod hosts within environmentally suitable niches. Despite their ubiquity, diversity, and potential ecological influence, Syndiniales are rarely considered in ecosystem models, and their role in energy transfer remains poorly understood. The role of infection requires further investigation, but overall, host mortality may fuel the microbial loop [11]. The observed dinospore distribution patterns suggest a strong interaction between parasite presence, host availability, and suitable environmental conditions that may facilitate transmission and the establishment of new infections.

## Supplementary Information

Below is the link to the electronic supplementary material.

Supplementary Material 1

## Data Availability

The raw sequence data can be found on the European Nucleotide Archive (ENA) website, under the project number: PRJEB101789. The original datasets, the environmental data, and RMarkdown documents for the data analysis are provided in GitHub and Zenodo online repositories: https://github.com/neeahanstrom/Syndiniales-dinospore-distributions; https://zenodo.org/records/21218935.
