# Supplementary material for: Environmental Gradients Shape the Distribution of Free-Living and Host-Associated Syndiniales Life Stages
Source: Microb Ecol. 2026 Jul 8;89:143. doi: 10.1007/s00248-026-02831-1 (PMC13350157; doi:10.1007/s00248-026-02831-1)
Supplement: Supplementary file 1 [file 248_2026_2831_MOESM1_ESM.docx]

**Environmental gradients shape the distribution of free-living and host-associated Syndiniales life stages**

**Neea Hanström, Kinlan M.G. Jan, and Monika Winder**

**Supplementary material**

**Supplementary figures**

**Supplementary Figure 1.** The vertical profiles of the environmental variables measured at each sampling location at the time of the sampling.

**Supplementary Figure 2.** Rarefaction curves of the samples after quality filtration. Crustacean reads were removed, and the samples with 100 or more reads were kept for analysis.

**Supplementary Figure 3**. ASV richness in the water samples at the different locations. (A) all water samples, (B) microplankton size fraction (>20 µm), and (C) pico-nano size fraction. (0.2-20 µm).

**Supplementary Figure 4.** Shannon and Gini-Simpson (1- D) diversity indices of the Syndiniales ASVs in the different filter sizes, sampling depths, and locations. Each point represents an individual sample.

**Supplementary Figure 5**. Relative read abundance of the Syndiniales groups in the water column at different locations and depth strata. A) Pico-nano (0.2-20 µm) size fraction, and B) Microplankton (>20 µm) size fraction. The letters on the x-axis stand for the sampling strata (S = surface, M = medium, and D = deep).

**Supplementary Figure 6.** PCoA based on Bray-Curtis dissimilarity of the Syndiniales clades across the filter sizes at the different locations and sampling strata. The colours represent the different sampling locations, and the shapes represent the filter size fractions.

**Supplementary tables**

**Supplementary Table 1.** The number of water filter samples that passed the data quality filtrations and were included in the analysis at each location and depth.

|  |  | Number of samples | | |
| --- | --- | --- | --- | --- |
|  | Depth | > 20 μm | 0.2-20 μm | Total |
| A17 | 0-30 | 2 | 2 | 4 |
|  | 30-60 | 2 | 2 | 4 |
|  | 60-90 | 2 | 0 | 2 |
| BY5 | 0-30 | 2 | 1 | 3 |
|  | 30-60 | 2 | 0 | 2 |
|  | 60-90 | 2 | 1 | 3 |
| BY15 | 0-30 | 2 | 2 | 4 |
|  | 30-60 | 1 | 2 | 3 |
|  | 60-90 | 2 | 2 | 4 |
| BY31 | 0-30 | 2 | 2 | 4 |
|  | 30-60 | 2 | 2 | 4 |
|  | 60-90 | 2 | 2 | 4 |
| Total |  | 23 | 18 | 41 |

**Supplementary Table 2.** The read number retrieved from the samples after the quality filtrations for the different Alveolata subdivisions. The columns with the (%) represent the percentage of the reads of each taxon out of the total Alveolata reads for each sample type (either water or zooplankton) or the total Alveolata reads.

|  | Water | Zooplankton | Total | Water (%) | Zooplankton (%) | Total (%) |
| --- | --- | --- | --- | --- | --- | --- |
| Alveolata | 419,558 | 89,329 | 508,887 | 82.4 | 17.6 | 100.0 |
|  |  |  |  |  |  |  |
| Dinoflagellata | 346,161 | 56,301 | 402,462 | 82.5 | 63.0 | 79.1 |
| Ciliata | 62,378 | 32,626 | 95,004 | 14.9 | 36.5 | 18.7 |
| Perkinsea | 10931 | 0 | 10,931 | 2.6 | 0.0 | 2.1 |
| Unclassified Alveolata | 88 | 402 | 490 | 0.0 | 0.5 | 0.1 |

**Supplementary Table 3.** Pairwise comparisons of ASV richness using Dunn’s post-hoc test with Benjamini–Hochberg (BH) adjustment to control for multiple testing. The significant combinations’ *p-*values are in bold.

| Comparison | Z-score | *p* unadj. | Adjusted *p*-value |
| --- | --- | --- | --- |
| A17 - BY15 | 2.31 | **0.0210** | **0.0420** |
| A17 - BY31 | 3.06 | **0.0022** | **0.0133** |
| A17 - BY5 | 2.31 | **0.0207** | **0.0420** |
| BY15 - BY31 | 0.72 | 0.4703 | 0.7055 |
| BY15 - BY5 | 0.19 | 0.8478 | 0.8478 |
| BY31 -BY5 | -0.47 | 0.6420 | 0.7704 |

**Supplementary Table 4.** PERMANOVAs of the Bray-Curtis dissimilarities based on the Syndiniales clade relative read abundance in the size fractions of the water samples across locations, depth strata, and the filter size fractions. The proportion of variance explained and *p-*values were used to evaluate the significance and importance of each vector (number of permutations 999). Significant *p -*values are presented in bold.

|  |  | Df | SumOfSqs | R2 | F-value | *p*-value |
| --- | --- | --- | --- | --- | --- | --- |
| Pico-nano |  |  |  |  |  |  |
| 0.2-20 μm |  |  |  |  |  |  |
|  | Location | 3 | 1.14 | 0.59 | 10.47 | **0.001** |
|  | Depth strata | 2 | 0.29 | 0.15 | 4.07 | **0.002** |
|  | Residual | 12 | 0.43 | 0.23 |  |  |
|  | Total | 17 | 1.91 | 1.00 |  |  |
|  |  |  |  |  |  |  |
| Micro |  |  |  |  |  |  |
| > 20 μm |  |  |  |  |  |  |
|  | Location | 3 | 1.68 | 0.67 | 14.97 | **0.001** |
|  | Depth strata | 2 | 0.23 | 0.09 | 3.03 | **0.022** |
|  | Residual | 17 | 0.64 | 0.25 |  |  |
|  | Total | 22 | 2.52 | 1.00 |  |  |
|  |  |  |  |  |  |  |
| All water samples |  |  |  |  |  |  |
| combined |  |  |  |  |  |  |
|  | Location | 3 | 2.16 | 0.42 | 11.90 | **0.001** |
|  | Depth strata | 2 | 0.17 | 0.03 | 1.40 | 0.18 |
|  | Size fraction | 1 | 0.92 | 0.18 | 15.15 | **0.001** |
|  | Residual | 34 | 2.06 | 0.40 |  |  |
|  | Total | 40 | 5.14 | 1.00 |  |  |

**Supplementary Table 5.** The relative importance and the significance of each environmental vector for the Bray-Curtis dissimilarity in the Syndiniales clade composition. Vectors were fitted to the PCoA ordination with multiple linear regression, and the significance was tested by permutation (number of permutations 999).

|  |  | PCoA 1 | PCoA 2 | R2 | *p* value |
| --- | --- | --- | --- | --- | --- |
| Pico-nano |  |  |  |  |  |
| 0.2-20 μm |  |  |  |  |  |
|  | Salinity | 0.274 | -0.962 | 0.69 | **0.005** |
|  | Oxygen | -0.200 | 0.980 | 0.61 | **0.011** |
|  | Temperature | -0.542 | -0.840 | 0.03 | 0.87 |
|  | Chlorophyll-a | -0.974 | 0.226 | 0.20 | 0.31 |
|  | Nitrate | 0.243 | -0.970 | 0.66 | **0.002** |
|  | Phosphate | 1.00 | 0.046 | 0.11 | 0.58 |
|  |  |  |  |  |  |
| Micro |  |  |  |  |  |
| > 20 μm |  |  |  |  |  |
|  | Salinity | 0.126 | -0.992 | 0.11 | 0.51 |
|  | Oxygen | 0.310 | 0.951 | 0.46 | **0.018** |
|  | Temperature | -0.970 | -0.245 | 0.16 | 0.36 |
|  | Chlorophyll-a | -0.576 | 0.818 | 0.01 | 0.95 |
|  | Nitrate | 0.020 | -1.000 | 0.17 | 0.28 |
|  | Phosphate | -0.076 | -0.997 | 0.05 | 0.72 |
